# Identification of two quantitative genes controlling soybean flowering using bulked-segregant analysis and genetic mapping

**DOI:** 10.3389/fpls.2022.987073

**Published:** 2022-11-30

**Authors:** Tianxiao Lv, Lingshuang Wang, Chunyu Zhang, Shu Liu, Jinxing Wang, Sijia Lu, Chao Fang, Lingping Kong, Yunlong Li, Yuge Li, Xingliang Hou, Baohui Liu, Fanjiang Kong, Xiaoming Li

**Affiliations:** ^1^ Guangdong Provincial Key Laboratory of Plant Adaptation and Molecular Design, Guangzhou Key Laboratory of Crop Gene Editing, Innovative Center of Molecular Genetics and Evolution, School of Life Sciences, Guangzhou Higher Education Mega Center, Guangzhou University, Guangzhou, China; ^2^ Key Laboratory of South China Agricultural Plant Molecular Analysis and Genetic Improvement, Guangdong Provincial Key Laboratory of Applied Botany, South China Botanical Garden, Innovative Academy of Seed Design, Chinese Academy of Sciences, Guangzhou, China; ^3^ University of Chinese Academy of Sciences, Beijing, China; ^4^ Suihua Branch Institute, Heilongjiang Academy of Agricultural Sciences, Suihua, Heilongjiang, China

**Keywords:** long-juvenile, BSA, positional cloning, *Tof11*, *FT*

## Abstract

Photoperiod responsiveness is important to soybean production potential and adaptation to local environments. Varieties from temperate regions generally mature early and exhibit extremely low yield when grown under inductive short-day (SD) conditions. The long-juvenile (LJ) trait is essentially a reduction and has been introduced into soybean cultivars to improve yield in tropical environments. In this study, we used next-generation sequencing (NGS)-based bulked segregant analysis (BSA) to simultaneously map qualitative genes controlling the LJ trait in soybean. We identified two genomic regions on scaffold_32 and chromosome 18 harboring loci *LJ32* and *LJ18*, respectively. Further, we identified *LJ32* on the 228.7-kb scaffold_32 as the soybean pseudo-response-regulator gene *Tof11* and *LJ18* on a 301-kb region of chromosome 18 as a novel *PROTEIN FLOWERING LOCUS T-RELATED* gene, *Glyma.18G298800*. Natural variants of both genes contribute to LJ trait regulation in tropical regions. The molecular identification and functional characterization of *Tof11* and *LJ18* will enhance understanding of the molecular mechanisms underlying the LJ trait and provide useful genetic resources for soybean molecular breeding in tropical regions.

## Introduction

Soybean [Glycine max (L.) Merr.], the main source of vegetable protein and oil globally, is a facultative short-day crop (GrahamVance, 2003). Flowering time and maturity traits significantly determine both plant adaptation to specific latitude and grain yield (Cober and Morrison, 2010; Zhong and Kong, 2022). Soybean is cultivated in a wide latitudinal range, from high-latitude areas such as Northeast China to tropical regions such as South America (Wang et al., 2016; Han et al., 2016). This broad ecological adaptability is enabled by genetic variation at major gene loci and quantitative trait loci (QTLs) controlling flowering and maturity (Lin et al., 2021a; Lin et al., 2021b). Multiple naturally occurring variants at these loci have become the targets of human selection and endow soybean with the flexibility to adapt to different areas with distinct photoperiod patterns. To date, 16 maturity loci, E1 to E11, J, Tof5, Tof11, Tof12, and LUX, have been identified by forward-genetic approaches (Bernard, 1971; Buzzell, 1971; McBlain and Bernard, 1987; Ray et al., 1995; Bonato and Vello, 1999; Cober and Voldeng, 2001a; Cober et al., 2010; Xia et al., 2012; Kong et al., 2014; Li et al., 2017; Samanfar et al., 2017; Wang et al., 2019; Lu et al., 2020; Bu et al., 2021; Dong et al., 2022). Among them, E1, E2, E3, E4, E7, E8, E10, Tof11, and Tof12 delay flowering and maturity under long-day (LD) conditions, and their recessive alleles enhance soybean adaptation to high latitudes (Cober and Voldeng, 2001b; Liu et al., 2008; Watanabe et al., 2009; Cober et al., 2010; Xia et al., 2012; Cao et al., 2017; Samanfar et al., 2017; Wang et al., 2019; Lu et al., 2020). A recent report indicated that the J protein associates with two LUX homologs to form the evening complex, which plays key roles in photoperiodic flowering and photoperiod sensitivity in soybean under both short-day (SD) and LD conditions (Bu et al., 2021). Tof11 and Tof12, encoding two homoeologous pseudo-response regulator (PRR) proteins, improved adaptation to the limited summer growth period at higher latitudes during soybean domestication (Lu et al., 2020).

At the other end of the latitudinal range, in the tropics, warm temperature and short photoperiod strongly induce rapid flowering and early maturity in photoperiod-sensitive soybean cultivars, making the vegetative phase very short and resulting in low yields (Parvez and Gardner, 1987; Destro et al., 2001). In these conditions, extension of the flowering and reproductive phases is necessary to allow greater vegetative growth and improve yield. The long-juvenile (LJ) trait has been introduced into tropical soybean cultivars to meet this need (Sinclair and Hinson, 1992; Carpentieri-Pípolo et al., 2002; Li et al., 2017; Lu et al., 2017). However, genetic information regarding this trait remains is limited. As the major classical locus conferring the LJ trait, J was identified as the ortholog of Arabidopsis thaliana EARLY FLOWERING 3 (ELF3). J depends genetically on the legume-specific flowering repressor E1 and directly downregulates E1 expression, thereby relieving the repression of two important FLOWERING LOCUS T (FT) genes (FT2a and FT5a) and promoting flowering under SD conditions (Lu et al., 2017; Fang et al., 2021). Recently, FT2a and FT5a were found to have variants of diverse origins that played distinctive roles as soybean spread to lower latitudes (Li et al., 2021). Tof16 was identified as a novel LJ locus that harbors the soybean homolog of the Arabidopsis LATE ELONGATED HYPOCOTYL (LHY), which delays flowering and improves yield at low latitudes (Dong et al., 2021). Loss of function of J, FT2a, or Tof16 is the major genetic base of soybean adaptation in tropical regions. Additionally, many QTLs associated with the LJ trait have been identified in soybean varieties (Fang et al., 2019; Lin et al., 2021a).

Conventional positional cloning and QTL mapping are powerful approaches for investigating the genetic control of phenotypic variation in agronomic traits (Burke et al., 2007). However, classical map-based gene cloning approaches are usually time-consuming owing to the need for genetic crossing and phenotypic analysis. As an alternative, the application of bulked segregant analysis (BSA) to QTL selection provides a simple strategy for rapidly identifying molecular markers tightly linked to the causal gene underlying a given phenotype (Giovannoni et al., 1991; Michelmore et al., 1991). BSA methods have been used in many organisms to map important genes (Mansur et al., 1993; Yi et al., 2006; Watanabe et al., 2011; Whipple et al., 2011). With the continuing advances in DNA sequencing technology, next-generation sequencing (NGS)-based BSA can dramatically accelerate the process of identifying causal genes of particular traits (Schneeberger and Weigel, 2011).

To investigate QTLs and corresponding candidate genes associated with the LJ trait, in this study we used genome-wide NGS-based BSA mapping of a soybean biparental population to identify two QTLs, named LJ32 and LJ18, conferring the LJ trait in soybean. We further validated these two QTLs and fine-mapped them by marker-based classical gene mapping to two intervals of 229 and 301 kb. Molecular and transgenic analyses demonstrated that the PRR gene Time of Flowering 11 (Tof11) and a PROTEIN FLOWERING LOCUS T-RELATED gene, Glyma.18G298800, may be responsible for the effects of the LJ32 and LJ18 loci. Overall, our study provides a useful genetic resource for soybean adaptation and molecular breeding to adapt soybeans for growth in tropical environments.

## Results

### Phenotypic analysis

**Figure 1:**
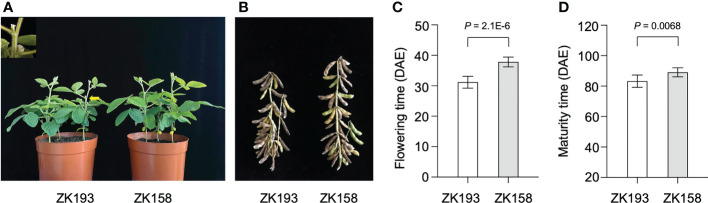
The phenotype of ZK193 and ZK158 under short-day (SD) conditions. **(A, B)** Flowering and maturity time of ZK193 and ZK158 under SD conditions. **(C)** Flowering time. Flowering time was recorded at the R1 stage. **(D)** Maturity time. Maturity time was recorded at the R8 stage. DAE (days after emergence). All data were given as mean ± SD (standard deviation, n = 10 plants). A Student’s t-test was used to generate the P values.

To identify additional loci contributing to the LJ trait, we developed a set of 213 recombinant inbred line (RIL) populations from a cross between two closely related soybean cultivars. We used the near-isogenic lines (NILs) ZK193 and ZK158, both with the genetic background of the Canadian cultivar Harosoy from L62-812, which have the same genotypes (e1/e2/E3/E4/E9/Dt1) for the major flowering time genes E1-E4 and E9 and the stem growth habit gene Dt1 (**Supplementary Table 1**). Nonetheless, ZK193 shows significantly earlier flowering and maturity than ZK158 under SD conditions (12 h light/12 h dark) (**Figure 1**). In addition, the two parents displayed different phenotypes in regard to several other traits, including node and pod number, grain number, and grain yield per plant (**Supplementary Figure 1**). To perform QTL mapping, we created cross combinations by pollinating ZK193 with pollen from ZK158 to develop descendant populations. We planted all RIL individuals and plants of both parent strains across 2 years (2018 and 2019) in Guangzhou, China, and recorded their flowering times at the R1 stage. The flowering data from 2018 and 2019 were strongly correlated (P < 0.01, R = 0.724), and we therefore used the data from 2019 for the following bulk segregation analysis (BSA) to detect flowering-associated loci.

### Analysis of flowering-associated loci by BSA and QTL mapping

**Figure 2:**
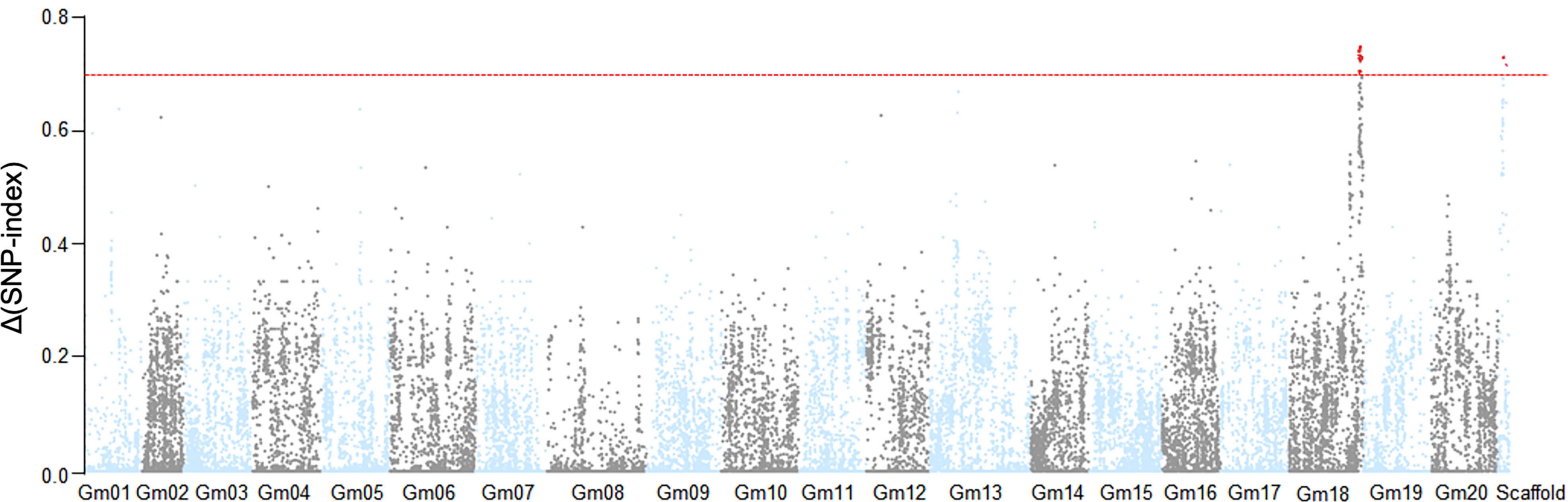
Identification of flowering time loci through SNP-index analysis. The result of SNP-index association analysis. The red lines show the threshold of Δ (SNP- index), which is represented by the top 5% of the permutation test. A larger value of Δ (SNP- index) indicates a stronger level of association. Under a threshold of 0.6964, two SNP markers on Gm18 and scaffold_32 significantly associated with the flowering time trait.

Based on the phenotypic assessment, we pooled genomic DNA from 30 individuals with extreme phenotypes (extremely early flowering and extremely late flowering) separately into an EF bulk sample and a LF bulk sample, respectively. We also extracted genome DNA from each parental line, isolated from leaves of 20 plants, for NGS-based BSA sequencing. After filtering, we identified clean reads and aligned them to the Williams 82 reference genome, and obtained high-quality single-nucleotide polymorphisms (SNPs) with which to calculate SNP-index values. We observed two peaks in the SNP-index plot, which we assigned as candidate flowering-time control regions in this population (**Figure 2**). The candidate regions were designated LJ trait 32 (LJ32) and LJ18 due to their locations on scaffold_32 and chromosome 18, respectively. SNP-index analysis revealed that the regions of the physical map around 229 kb on scaffold_32 and 4.7 Mb on chromosome 18 might be associated with flowering time (**Supplementary Table 2**, **Supplementary Figure 2**).

Scaffold_32 is a fragment that failed to be successfully assembled in the Williams 82 reference genome. Because it is a small fragment (229 kb), we performed ANOVA analysis to confirm the flowering-associated interval on Scaffold_32 (**Supplementary Table 2**). The results showed that the flowering times of all plants with theZK193 marker pattern for Scaffold_32 were significantly earlier than the mean value for plants with the ZK158 pattern. We inferred that a QTL is present in the same chromosome region as the marker Tof11, here named LJ32. Under SD conditions, LJ32 significantly affected flowering time, as shown by genome-wide analyses with permutation tests (P < 0.05) (**Figure 2**). To validate the candidate region chromosome 18 identified by BSA mapping, we used numerous insertion-deletion (Indel) and SNP markers in the region that are polymorphic between the two parental lines to genotype and constructed genetic linkage maps in the RIL population using the Kosambi function. A total of 11 markers, spanning 67.8 cM (**Supplementary Figure 2**), covered a part of the region of linkage group 18. The main marker type contributing to this linkage map was Indel markers, while the linkage gaps between the Indel markers were bridged by SNP markers. The constructed map was generally consistent with the US Department of Agriculture soybean genetic linkage map (Choi et al., 2007). QTL analyses revealed that LJ18 was located in a region between markers ID181191 and ID181120 on chromosome 18 (**Supplementary Table 2**). Two significant QTLs for flowering time, LJ32 and LJ18, were consistently detected in the 2018 and 2019 data, validating the accuracy of the NGS mapping results.

### Characterization of LJ32

On the Scaffold_32 fragment, 22 genes were annotated. Of these, Glyma.U034500, encoding a PRR family protein, was identified as Tof11, which has been reported to delay flowering in LD conditions (16 h light/8 h dark) (Lu et al., 2020) (**Supplementary Table 3**). Upon further analysis of the NGS data, we identified a 1-bp deletion (A2210-) in the last exon of Tof11, causing frameshifts and premature termination of protein translation, in the parent ZK193 compared with that in ZK158 (**Supplementary Figure 3**). Previous study found that this Tof11 haplotype, which we named Tof11-1, was the most abundant in landraces and improved cultivars and identified to be selected at an early stage of modern soybean breeding, and Tof11 is genetically dependent on E1 (Lu et al., 2020). We evaluated the effect of Tof11 on transcriptional regulation of E1 under SD conditions, A similar result was obtained in the parents showing that functional alleles of Tof11 in ZK158, relative to the respective mutant alleles in ZK193, increased E1 expression (**Supplementary Figure 4**). We thus identified Tof11 as a candidate gene potentially responsible for the effect of LJ32.

**Figure 3:**
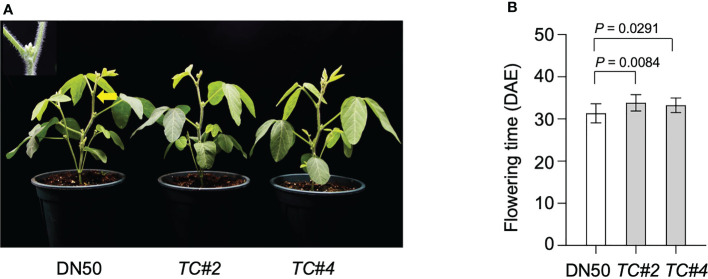
Confirmed identity of LJ32 by transgenic complementation. **(A)** The flowering phenotype of two independent transformants of complementation, TC#2 and TC#4, and the control, DN50 Tof11-1 under SD (12 h light/12 h dark). Scale bar, 10 cm. **(B)** Flowering time. All data were given as mean ± SD (standard deviation, n = 10 plants). A Student’s t-test was used to generate the P values.

To characterize the function of Tof11 in soybean LJ regulation, we grew two complementary transgenic Tof11 lines (TC#2 and TC#4), along with a wild-type (WT) cultivar Dongnong 50 (Lu et al., 2020), under SD conditions. The TC#2 and TC#4 plants flowered slightly but significantly later than WT plants under SD conditions (**Figure 3**), supporting our hypothesis that LJ32 is encoded by TOF11.

**Figure 4:**
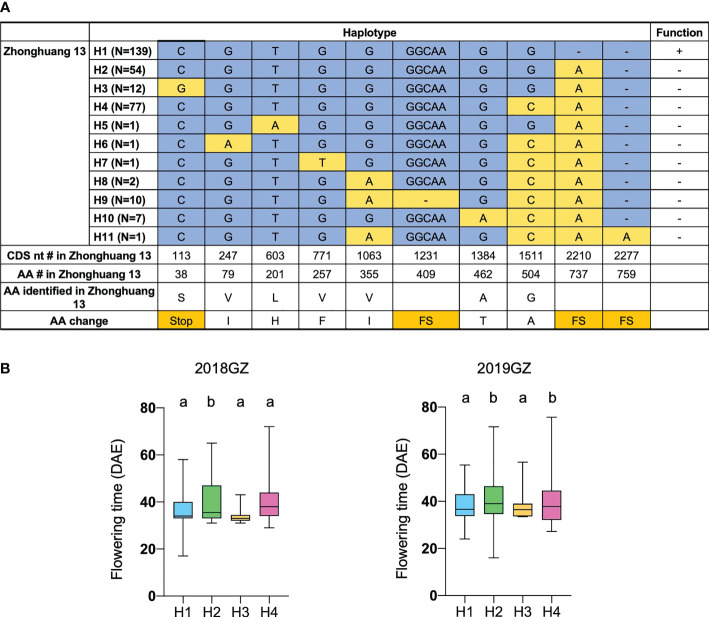
Flowering time of different alleles of Tof11. **(A)** Haplotypes and their origins of Tof11. FS, frameshift. **(B)** Flowering time of eleven haplotypes of Tof11 from the 338-accession panel. 20GZ, the accessions were planted in Guangzhou, China, in 2020; 18GZ, planted in Guangzhou, China, 2018. Two-tailed, two-sample t-test were used to generate the P values. Data represent mean ± SD of three biological replicates. Different lowercase letters represent significant differences at the level of P < 0.05, based on the Student’s t test.

To further validate the function of Tof11 in soybean LJ trait regulation under SD and identify the allelic variations of Tof11, we looked for variations in the Tof11 coding sequence in our collection of 338 re-sequenced soybean accessions from low-latitude regions grown in Guangzhou (Li et al., 2021). We identified 11 haplotypes, of which H2 and H4 (functional alleles) resulted in significantly later flowering than H1 and H3 (loss-of-function alleles) (**Figure 4**). The remaining alleles were not assessed because they were found in only a few accessions (**Figure 4**). Notably, in these 338 accessions, the frequency of functional alleles (48%) was similar to that of loss-of-function alleles, indicating that variations in Tof11 may contribute to the geographic distribution of soybean accessions in lower-latitude regions. Together, our observations indicated that Tof11 is the most likely causal gene in the LJ32 locus for the LJ trait.

### Positional cloning of LJ18

**Figure 5:**
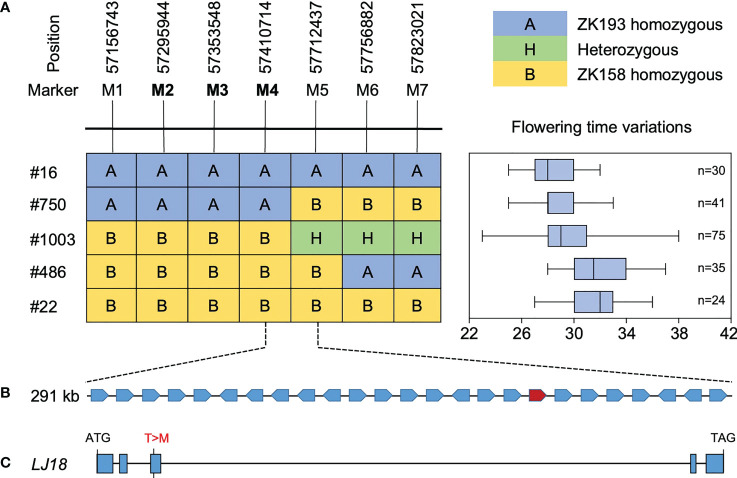
Identification of the LJ18 gene by map-based cloning. **(A)** Segregation of flowering time and delimitation of the LJ18 locus to a 301-kb region between the marker M4 and M5 on chromosome 18. A, homozygous for the allele from ZK193; B, homozygous for the allele from ZK158; H, heterozygous. Segregation of flowering time is shown in right box-plot format, where the interquartile region, median, and range are represented by the box, the bold vertical line, and the horizontal line, respectively. Each box plot corresponds to the segregant on the same row in left. **(B)** The delimited region contains 25 predicted genes. **(C)** Allelic variation in the LJ18 candidate gene Glyma.18G188800 between ZK193 and ZK158.

To further delineate the LJ18 locus, we surveyed the genotypes at two markers within the QTL in 1354 plants segregated from heterozygous plants and detected five recombinants. We also investigated the segregation pattern in the residual heterozygous lines (RHLs) (**Supplementary Figure 5**). Fine-mapping with seven additional molecular markers delimited the LJ18 genomic region to an ~301-kb region between markers M4 and M5 (**Figure 5**), which harbors 25 genes according to the Williams 82 reference genome (**Supplementary Table 4**). Among them, three PROTEIN FLOWERING LOCUS T-RELATED genes were annotated: Glyma.18G298800, Glyma.18G298900 (GmFT1a), and Glyma.18G299000 (GmFT1b) (**Supplementary Figure 6**). We found no variants in GmFT1a resulting in amino acid changes and one non-synonymous SNP each in GmFT1b and Glyma.18G298800 (LJ18) (**Supplementary Figure 6**, **7A**).

GmFT1a and GmFT1b are widely recognized as FLOWERING LOCUS T (FT) homologs in soybean (Kong et al., 2014), and variants in promoter regions can regulate gene expression and function (Liu et al., 2018; Li et al., 2021). We therefore tested the functions of each gene in soybean flowering regulation using loss-of-function mutants generated by CRISPR/Cas9 gene editing in the Williams 82 cultivar. We obtained multiple mutants for both GmFT1a and GmFT1b (**Supplemental Figures 7B, C**). Unexpectedly, phenotypic analysis detected no significant difference in flowering time between the Williams 82 and these single mutants under SD conditions (**Supplementary Figure 7D**).

**Figure 6:**
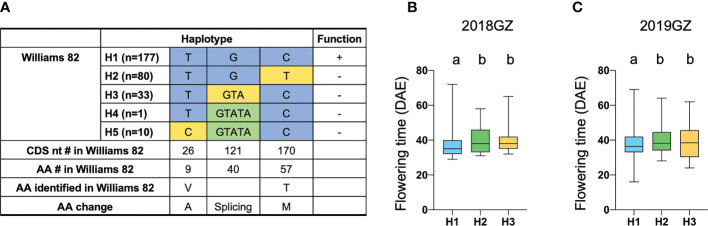
Flowering time variations of different alleles in LJ18. **(A)** Haplotypes and their origins of LJ18. **(B, C)** Variation of flowering time of LJ18 haplotypes from the 338-accession panel. 18GZ, the accessions were planted in Guangzhou, China, in 2018; 19GZ, planted in Guangzhou, China, 2019. Two-tailed, two-sample t-tests were used to generate the P values. Data represent mean ± SD of three biological replicates. Different lowercase letters represent significant differences at the level of P < 0.05, based on the Student’s t test.

Another FT-related protein, encoded by Glyma.18G298800, was annotated and located in tandem with GmFT1a and GmFT1b on chromosome 18 (**Supplementary Table 4**). NGS data revealed a non-synonymous SNP in the 3rd exon specifying a polymorphism between the two parental strains at residue T170C (T57M), which is threonine (T) in ZK158 but methionine (M) acid in ZK193 (**Figure 5C**). These observations suggested that Glyma.18G298800 is a probable candidate for the causative gene in the LJ18 locus. We investigated the allelic variation of Glyma.18G298800 using the same strategy described above for Tof11 and identified five haplotypes, among which haplotype 2 (H2) corresponded to the lj18 allele (**Figure 6A**). we examined the geographic distribution of various alleles at LJ18 loci within the 338 accessions including the five LJ18 alleles (**Figure 6A**). All alleles showed no significant geographical distribution characteristics (**Supplementary Table 5**). We also analyzed the association of the Glyma.18G298800 haplotypes with flowering time in Guangzhou over 2 years. Accessions carrying H2 and H3 flowered significantly later than those carrying H1 (**Figure 6B, C**). This observation indicated that the polymorphism at nucleotide 170 in Glyma.18G298800 may lead to the variation in flowering time, supporting a role for Glyma.18G298800 in the control of flowering time in diverse genetic backgrounds. Taking these results together, we suggest that Glyma.18G298800 is a likely candidate gene for LJ18 and influences flowering regulation under SD conditions.

### Functional analysis of Glyma.18G298800 in soybean flowering regulation

**Figure 7:**
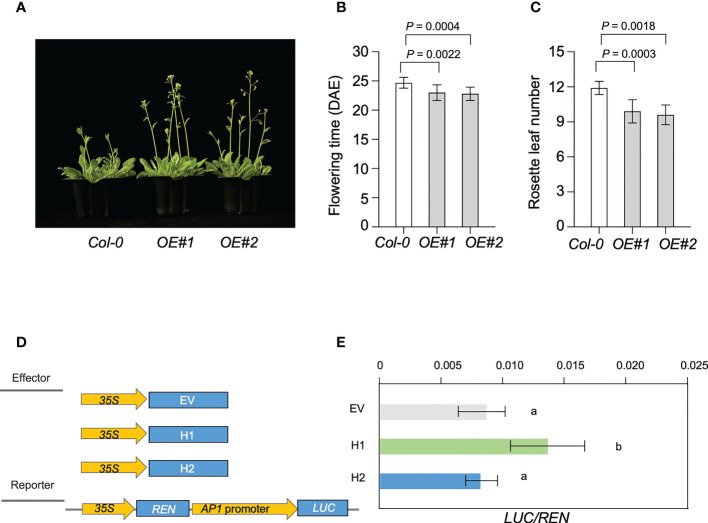
Genetic analysis of LJ18. **(A)** Flowering phenotype of the transgenic plants of Arabidopsis overexpressing LJ18, OE#1 and OE#2 relative to the untransformed control Col-0 plants. **(B)** Flowering time of Col-0, and LJ18-overexpressing plants under long day (LD) conditions at 22°C. **(C)** The number of rosette leaves in at least 10 plants at bolting was used as an indicator of flowering time. Statistically significant differences are indicated by different lowercase letters (Student’s t test, P < 0.05). **(D)** Schematic maps of the constructs used in the transient expression assay. Empty vector (EV) was used as the negative control. Promoter of AP1a was separately inserted into the vector to get the reporters. 35S, CaMV35S promoter; LUC, firefly luciferase; REN, Renilla luciferase. **(E)** Transcriptional activity of AP1a promoter reporters in N. benthamina. REN was used as an internal control. LUC/REN represented the transcriptional activity of AP1a promoter. Data represent mean ± SD of three biological replicates. Different lowercase letters represent significant differences at the level of P < 0.05, based on the Student’s t test.

To confirm the expression patterns of Glyma.18G298800, we used reverse transcription quantitative PCR (RT-qPCR) to analyze the expression patterns of Glyma.18G298800 in leaves at different development stage of Williams 82 soybean (**Supplemental Figure 8A**). We found that Glyma.18G298800 transcripts were much more abundant in cotyledons and leaves than in other tissues. To preliminarily examine the function of Glyma.18G298800, we ectopically expressed Glyma.18G298800 in the Arabidopsis Columbia-0 (Col-0) ecotype. Among the resulting transgenic lines, RT-qPCR results showed that two independent lines exhibited relatively higher Glyma.18G298800 transcript levels than Col-0, and we selected these for further phenotypic analysis (**Supplemental Figure 8B**). The results showed that overexpression of Glyma.18G298800 significantly promoted flowering time compared with that of Col-0 (**Figures 7A–C**).

Next, we explored the molecular mechanism underlying the effect of Glyma.18G298800 on the LJ trait through a dual-luciferase assay. As soybean APETALA1 (AP1) is reported to be the primary target for FT regulation (Chen et al., 2020; Li et al., 2021), we used this assay to examine the effect of Glyma.18G298800 on AP1a, by fusing a 3-kb fragment of the AP1a promoter to the luciferase (LUC) reporter gene. We used Glyma.18G298800H1 and Glyma.18G298800H2 driven by the CaMV 35S promoter as effectors. We transformed each effector construct, together with the reporter construct, into N. benthamiana. Compared with the reporter vector control, the Glyma.18G298800H1 effector promoted the activity of AP1a promoter, as revealed by an increased LUC/REN (**Figure 7D, E**). The Glyma.18G298800H2 effector resulted in a lower LUC/REN ratio than Glyma.18G298800H1 effector, indicating that Glyma.18G298800H2 has no effect on the activity of the AP1a promoter (**Figure 7D, E**), which is consistent with the parental flowering phenotypes and with the results of the haplotype analysis above. Collectively, our results demonstrated that the PROTEIN FLOWERING LOCUS T-RELATED gene Glyma.18G298800 may function as a flowering promotor in soybean.

## Discussion

In tropical regions, days are short during the growing season, neither temperature nor water are limiting, and the long-juvenile (LJ) trait is well established as an important adaptation that allows the crop to take full advantage of this favorable environment (Li et al., 2021). The introduction of the LJ trait in the 1970s overcame limitations on soybean growth, allowing its production to be extended to lower-latitude (tropical) areas (Neumaier and James, 1993; Carpentieri-Pípolo et al., 2002). For example, until 1960, soybean cultivars used in Brazil were imported from the United States, and cultivation areas were limited to above 22 degrees south latitude. In recent decades, however, the introduction of LJ germplasm has enabled Brazil to become the world’s second-largest soybean producer (Neumaier and James, 1993). Notwithstanding the importance of LJ genes for soybean adaptation and yield in tropical regions, however, the underlying genetic basis and the trajectory of adaptation to low latitudes by means of these genes have remained largely unknown.

In this study, we developed a hybrid population from crosses between the NILs ZK193 and ZK158. NGS-based BSA combined with QTL analysis revealed two QTLs associated with the LJ trait, LJ32 and LJ18, located on scaffold_32 and chromosome 18, respectively, in the Williams 82 reference genome. Scaffold_32 is a 229-kb fragment that is assembled on chromosome 11 of the Zhonghuang 13 reference genome (Lu et al., 2020). We considered the gene Tof11, located in this region, as a candidate for the LJ32 locus. Although Tof11 is reported to delay flowering under LD conditions and to have contributed to ancient flowering-time adaptation (Lu et al., 2020), its function in LJ regulation had not previously been investigated. Here, we demonstrated that Tof11 functions as a flowering inhibitor under SD conditions and regulates the soybean LJ trait at low latitude.

FT proteins comprise a clade of the plant phosphatidylethanolamine-binding protein (PEBP) family and act as highly conserved regulators that are pivotal in the flowering pathways of various crop species (Kong et al., 2010). In soybean, several FT homologs have been reported as candidate florigens or antiflorigens under LD or SD conditions, including GmFT2a, GmFT5a, GmFT1a, and GmFT4 (Zhao et al., 2016; Liu et al., 2018; Jiang et al., 2019; Lu et al., 2020). Furthermore, GmFT2a and GmFT5a act as floral promoters conferring the LJ trait and played distinct roles as soybean spread to lower latitudes (Nan et al., 2014; Cai et al., 2020; Li et al., 2021; Yue et al., 2021). Here, we report that Glyma.18G298800, a novel FT homolog, is a possible candidate for the LJ18 locus that regulates the soybean LJ trait. This finding provides preliminary evidence that Glyma.18G298800 may contribute to delaying the flowering time of soybean varieties by inhibiting AP1a expression.

In summary, we used NGS-based BSA combined with QTL analysis to reveal two different QTLs conferring the LJ trait. We identified LJ32 as the soybean PRR gene Tof11 and LJ18 as the PROTEIN FLOWERING LOCUS T-RELATED gene Glyma.18G298800. The natural variants of both genes have significant influence on flowering time in SD accessions, suggesting that these two genes may play important roles in controlling flowering time in tropical regions. The identification and characterization of these LJ-related genes will contribute to the understanding of the genetic and molecular mechanisms underlying the LJ trait and could be used to ensure the successful deployment of high-yield germplasm in tropical environments.

## Materials and methods

### Plant materials, growth conditions, and phenotyping

The NILs lines, ZK193 and ZK158 with genetic background of Canadian cultivar Harosoy from L62-812 were used; they have the same maturity genotypes at E1, E2, E3, and E4 (e1/e1 e2/e2 E3/E3 E4/E4 DT1/DT1) (**Supplementary Table S1**). The cross combinations were made by pollinating ZK193 with pollen from ZK158 to develop RIL populations that were used for (NGS)-based Bulked segregation analysis (BSA) (**Supplementary Table S2**). The populations and low-latitude-adapted accessions for phenotyping were grown under naturally SD conditions (12 h light/12 h dark) in the field from 2017 to 2018 at the experimental station of Guangzhou University, Guangzhou (22° 26′ N, 112° 57′ E). The transformants for phenotyping were sown in pots in growth cabinets under SD conditions (12 h light/12 h dark)

Days to flowering were recorded at the R1 stage (first open flower appeared) for each plant47. The R1 values reported for the parents and populations are means from 10 plants. The number of parents and RIL plants used in each experiment are listed in **Supplementary Table 2**. Days to flowering (R1) were individually recorded and subjected to analysis of variance. Means of days to flowering among lines were compared with Tukey’s HSD test using the Statistica software 03J (StatSoft). Plant height, number of branches, number of nodes, number of pods per plant, number of grains per plant, and yield per plant were all recorded at the R8 stage. All data are given as means ± s.e.m. (n = 10 plants). Two-tailed, two-sample t-tests were used to generate the P values.

### DNA bulks construction and illumina sequencing

BSA was used to group the RIL population and its parents, Two DNA bulks for sequencing were first made by selecting extreme individuals from the 213 RIL population plants with the basic statistics of the phenotypic data. One pool for early flowering comprised 30 lines with early flowering time and the other pool for late flowering involved 21 lines. DNA was extracted individually from leaves of plants, using a genomic DNA purification kit (Thermo Fisher Scientific Inc., United States) according to the manufacturer’s protocol. The GC content, repeated sequences, and genetic characteristics of the DNA pools were analyzed by Biomarker (Beijing, China). About 20 μg of DNA from the two bulks and two parental lines were used to construct paired-end sequencing libraries, The genomic DNA pools were digested using the XhoI and MseI restriction enzymes (NEB, Ipswich, MA, USA), followed by PCR amplification, fragment amplification, fragment selection, fragment extraction and amplification, and fragment sequencing using the Illumina HiseqTM 2500 (Illumina, Inc; San Diego, CA, USA) at Biomarker Technologies. Real-time monitoring was performed for each cycle during sequencing and the ratio of high-quality reads with quality scores greater than Q30 (a quality score of 30, indicating a chance of 0.1% for an error and thus 99.9% confidence) in the raw reads GC content was calculated for quality control.

After removing adapter and low-quality reads, the clean reads were further rechecked for quality control using FASTQC1. High-quality sequences were aligned and to the Glycine max Wm82.a2.v1 reference genome from Phytozome2 (https://phytozome-next.jgi.doe.gov) using BWA with default parameters (Langmead and Salzberg, 2012).

### SNP-index association analysis

GATK (Genome Analysis Toolkit) was used to call SNPs and small indels across parental lines and bulks (McKenna et al., 2010). The relative marker abundance in bulked DNA pool 1 (the early flowering pool) was calculated as the number of reads of the paternal allele divided by the total of reads which then gives proportion paternal alleles (or alternatively maternal alleles), whereas in pool 2 (the late flowering pool), Homozygous SNPs between parental lines and high-quality SNPs (minimum sequence read depth: 10 with SNP base quality ≥ 100 in pools) were selected for SNP-index analysis. A SNP-index was calculated at each SNP position for both pools using the base in parental lines as alternative base (Abe et al., 2012; Takagi et al., 2013b). Thus, the SNP-index was assigned as 0 or 1, when entire short sequence reads contained genomic fragments derived from parental lines, respectively. A Δ(SNP-index) was calculated by subtraction of the early flowering index from the late flowering index (Fekih et al., 2013; Takagi et al., 2013a). Thus, a high Δ(SNP-index) value of a SNP locus is indicative of an allele that was both very frequent in the pool 1and depleted in the pool 2.

### QTL identification and statistical analysis

The polymorphisms between the parents introduced two kinds of markers, SNP and Indel. Markers were developed based on re-sequencing data from the parents, ZK193 and ZK158. The whole genome re-sequencing of ZK193, ZK158 and the Indel analysis using the software of SOAPindel was conducted by BGI-Shenzhen, China as described previously (Kong et al., 2014). The procedures for polymerase chain reaction and gel-electrophoresis were adopted as reported earlier (Li et al., 2017). Marker order and distance were determined by Map Manager Program QTXb20 using the Kosambi function and a criterion of 0.001 probability (d.f. = 1) and a genetic map was constructed (Lu et al., 2015). Mapchart 2.1 was used to draw the linkage groups (Voorrips, 2002). The Multiple QTL Model (MQM), implemented by MapQTL 5.0 was used for QTL detection (Van Ooijen, 2004). A LOD score of 2.5 was used as a minimum to declare the significance of a QTL in a particular genomic region. The tests of 1000 permutations at a 0.05 probability were conducted to identify the genome-wide LOD score (Churchill and Doerge, 1994).

### Resequencing and variation calling

The resequencing data, VCF files and flowering time data from the 338-accession panel used in this study were obtained from Li et al. (Li et al., 2021). The VCF files were processed using the VCFtools software (v.0.1.16). Paired-end resequencing reads of the 338 accessions were mapped to the Glycine max Wm82.a2.v1 (https://phytozome.jgi.doe.gov/pz/portal.html#!info?alias=Org_Gmax) with BWA software (Version: 0.7.17-r1188, http://bio-bwa.sourceforge.net/) with the default parameters. The duplicates of sequencing read for each accession were filtered with the Picard package (Version: V1.109, http://broadinstitute.github.io/picard/), and uniquely mapping reads were retained in BAM format. Reads around indels from the BWA alignment were realigned with the IndelRealigner option in the Genome Analysis Toolkit (GATK, Version: V3.2-2, https://gatk.broadinstitute.org/hc/en-us). SNP and indel calling were performed with GATK and SAMtools software (Version: 1.9, http://samtools.sourceforge.net/). SNPs with MAFs less than 1% were discarded, and indels with a maximum length of 20 bp were included. SNP annotation was carried out based on the Williams82 genome, with Annovar (https://annovar.openbioinformatics.org/en/latest/).

### Fine Mapping of LJ18 locus

For genetic analysis, we surveyed the genotypes by two markers for the F4 populations and conducted a segregating-heterozygous inbred family (n = 1354) that was heterozygous at LJ18 locus. The segregation pattern was carefully observed in the residual heterozygous lines (RHLs), in which the segregation occurred only at LJ18 locus, seven additional Indel and SNP markers between markers ID181191 and ID181120 were identified (**Figure 5A**; **Supplementary Table S5**). Three recombinants in the region between M1 and M7 were genotyped using four Indel markers and three SNP markers (bold) (**Figure 5A**), and the flowering time of their progenies were evaluated to delimit the genomic interval containing LJ18. The genotypes of the LJ18 allele were analyzed by tagging marker M4 or M6 (**Supplementary Table S5**). The LJ18 allele was genotyped by its functional markers.

### Plasmid construction and plant transformation

The CDS of the LJ18 candidate gene Glyma.18G298800 were obtained from ZK193 and ZK158. The CDS fragments were amplified by overlapping PCR to obtain one fragment and then introduced into the PTF101-Gene vector (containing the bar gene for glufosinate resistance) (Li et al., 2021). The construct (PTF101-35S:LJ18) was next introduced into the Agrobacterium tumefaciens strain EHA105, and 35S:LJ18-3flag transgenic lines were obtained through A. tumefaciens mediated transformation using the floral dip method in Col-0 wild-type followed by screening with 1/500 10% (w/v) basta. The FT1b knockout construct was produced by CRISPR-Cas9 as described previously (Ma et al., 2015). Two 20-bp sequences in the exons of FT1a and FT1b were selected as target sites for Cas9 cleavage (**Supplementary Figure S6**). Primers used for plasmid construction are listed in **Supplementary Table S6**. The above-mentioned CRISPR-Cas9 plasmid was transformed into Williams82 plants, and the transgenic plants were selected by basta (Ingbio, Lot: CB26213210).

### Gene expression analysis

Soybean seedlings grown under SD conditions were harvested from the leaf of V3-stages for total RNA extraction using E.Z.N.A. Total RNA Kit I (Omega) and reverse transcribed to cDNA using MMLV-Reverse Transcriptase (Promega). qPCR was performed using a LightCycler 480 thermal cycler system (Roche) with KAPA SYBR Fast qPCR Kit Master Mix (Kapa Bio). The difference between the cycle threshold of target genes and the cycle threshold of the control gene was calculated by the relative quantification method (2-△△Ct) and used to evaluate quantitative variation (Livak and Schmittgen, 2001). All expression analyses were performed with at least three biological replicates (three replicates of samples were taken from the same batch of plants, and total RNA was extracted from the pooled three to five tissues per independent replicate). The above experiments were independently performed at least three times, and representative results are shown. The primers used for gene expression analysis are listed in **Supplementary Table S6**.

### Transient expression assays

To generate the AP1a pro-LUC reporter construct, ~3 kb AP1a promoter was cloned into the pGreenII0800-LUC vector (Li et al., 2021). The Renilla Luciferase (REN) gene under the control of 35S promoter in the pGreenII0800-LUC vector was used as the internal control. The coding regions of 35Spro: LJ18-H1 and 35Spro : LJ18-H2 were cloned into the pGreenII62-SK vector and used as effectors. All primers used for these constructs are listed in **Supplementary Table S6**. The A. tumefaciens mixtures were infiltrated into three leaves of tobacco plants as described previously (Yue et al., 2021). p35S-LJ18-H1-LUC and p35S-LJ18-H2-LUC represent the A. tumefaciens carrying the effector constructs and the control vector pGreenII 0800-LUC. The tobacco leaves were allowed to recover for 48 h. LUC/REN activity showing the results from three independent replications and the value of each replication were represented by a dot. The LUC and REN activities were measured using the Dual-Luciferase Reporter Assay System (Promega) under the manufacturers’ instructions. The LUC/REN ratio was presented with three biological replicates.

### Data availability

For phenotypic evaluation, at least ten individual plants were analyzed per accession, and the exact number of individuals (n) are presented in all figure legends. The exact number of replicates is given in figure legends. Mean values for each measured parameter were compared using one-way analysis of variance from SPSS (version 20, IBM) or one-tailed, two-sample Student’s t-tests from Microsoft Excel, whenever appropriate; the statistical tests used for each experiment are given in the figure legends. Whole-genome sequencing data for ZK193, ZK158, and the two bulks are deposited at CNCB-NGDC and are publicly available as of the date of publication. Any additional information required to reanalyze the data reported in this paper is available from the lead contact upon request.

## Data availability statement

The data presented in the study are deposited in the “Bioproject” repository accession number “PRJNA896173”.

## Author contributions

XL, FK, and BL designed the experiments, supervised the study, and managed the projects. TL, LW, CZ, and ShL. performed most of the research. JW, SiL, CF, LK, YunL, YugL, and XH performed data analysis. TL, CZ, SL, and XL drafted and revised the manuscript. All authors contributed to the article and approved the submitted version.

## Funding

This work was funded by the National Natural Science Foundation of China (31801390, 32090064, 31725021, 32201800). This work was also supported by Major Program of Guangdong Basic and Applied Research (2021A1515110522), Guangdong Provincial Key Laboratory of Plant Adaptation and Molecular Design (2022B1212010013-3) and Hainan Yazhou Bay Seed Lab of China (B21HJ0110).

## Acknowledgments

We would like to acknowledge Dr. Shulin Liu for the data analysis.

## Conflict of interest

The authors declare that the research was conducted in the absence of any commercial or financial relationships that could be construed as a potential conflict of interest.

## Publisher’s note

All claims expressed in this article are solely those of the authors and do not necessarily represent those of their affiliated organizations, or those of the publisher, the editors and the reviewers. Any product that may be evaluated in this article, or claim that may be made by its manufacturer, is not guaranteed or endorsed by the publisher.

## Supplementary material

Supplementary Figure 1 Yield related traits of the parent lines. **(A–F)** Plant height, number of nodes, number of branches, total pods per plant, total seeds per plant, and 100 seeds weight of ZK193 and ZK158. The plants were grown in standard field under artificially controlled SD (12 h light/12 h dark). All data are given as mean ± s.e.m. (n = 10 plants). Two-tailed, two-sample *t*-tests were used to generate the *P* values.

Supplementary Figure 2 Linkage groups containing QTLs for flowering time found in RIL populations. The Linkage groups containing QTLs for flowering time found in RIL populations. The title of linkage groups followed by the chromosome number in parenthesis is indicated at the top. The genetic distance of markers (cM) from the top of each linkage group are given on the left-hand side. The arrows indicate the position of the QTL peaks.

Supplementary Figure 3 Protein sequence alignment of *Tof11* alleles. Similarity of the predicted amino acid sequence of Tof11 homologs. The sequences were extracted from the Zhonghuang 13 genome database.

Supplementary Figure 4 The expression level of *E1* in ZK193 and ZK158. Expression level of *E1* in the NILs. Soybean *Tublin* was used as an internal control. Data are means ± s.e.m (n = 3). A Student’s *t*-test was used to generate the *P* values.

Supplementary Figure 5 Frequency distribution of flowering times in *LJ18* residual heterozygous lines (RHL) populations.

Supplementary Figure 6 Protein sequence alignment of FT homologs. Similarity of the predicted amino acid sequences of FT homologs in soybean. The sequence of *LJ18* (Glyma.18G298800), *FT1a* (Glyma.18G298900), *FT1b* (Glyma.18G299000), *FT2a* (Glyma.16G150700), *FT2b* (Glyma.16G151000), *FT3a* (Glyma.16G044200), *FT3b* (Glyma.19G108100), *FT4* (Glyma.08G363100), *FT5a* (Glyma.16G044100), *FT5b* (Glyma.19G108200), and *FT6* (Glyma.08G363200) were obtained from the Williams 82 genome database. Highly conserved amino acids are in dark blue, blue, and light blue depending on the level of identity (darker = higher level). The light-yellow arrows indicate the Phosphatidyl Ethanolamine-Binding Protein (PEBP) domain (predicted by NCBI CD-Search: https://www.ncbi.nlm.nih.gov/Structure/cdd/wrpsb.cgi ). The red box represents the site of core amino acid replacement in the PEBP domain of lj18.1_H2.

Supplementary Figure 7 Plant material for experiments. **(A)** The predicted amino acid sequence of Glyma.18G299000 (*FT1b*) in ZK193 and ZK158, obtained from the Williams 82 genome database**. (B, C)** Characterization of *ft1a* and *ft1b* mutants without *Cas9* gene in the T_2_ generation. Sequences of WT and mutant plants at target sites. Dashes indicate deleted nucleotides. Nucleotides in red indicate PAM. Red arrowheads indicate mutation locations. **(D)** Flowering time of Cas9-FT1a and Cas9-FT1b under SD (12 h light/12 h dark) conditions.

Supplementary Figure 8 Expression pattern of *Glyma.18G298800.* **(A)** Expression of *Glyma.18G298800* in difference tissues of Williams82 under SD. Soybean *Tublin* was used as an internal control. Data are means ± s.e.m (n = 3). **(B)** Expression level of *Glyma.18G298800* in the transgenic lines of Arabidopsis. OE, overexpressing. *Arabidopsis UBQ10* was used as an internal control. Data are means ± s.e.m (n = 3).
