# Supplementary figures and images for: Identification of two quantitative genes controlling soybean flowering using bulked-segregant analysis and genetic mapping
Source: Front Plant Sci. 2022 Nov 30;13:987073. doi: 10.3389/fpls.2022.987073 (PMC9749486; doi:10.3389/fpls.2022.987073)

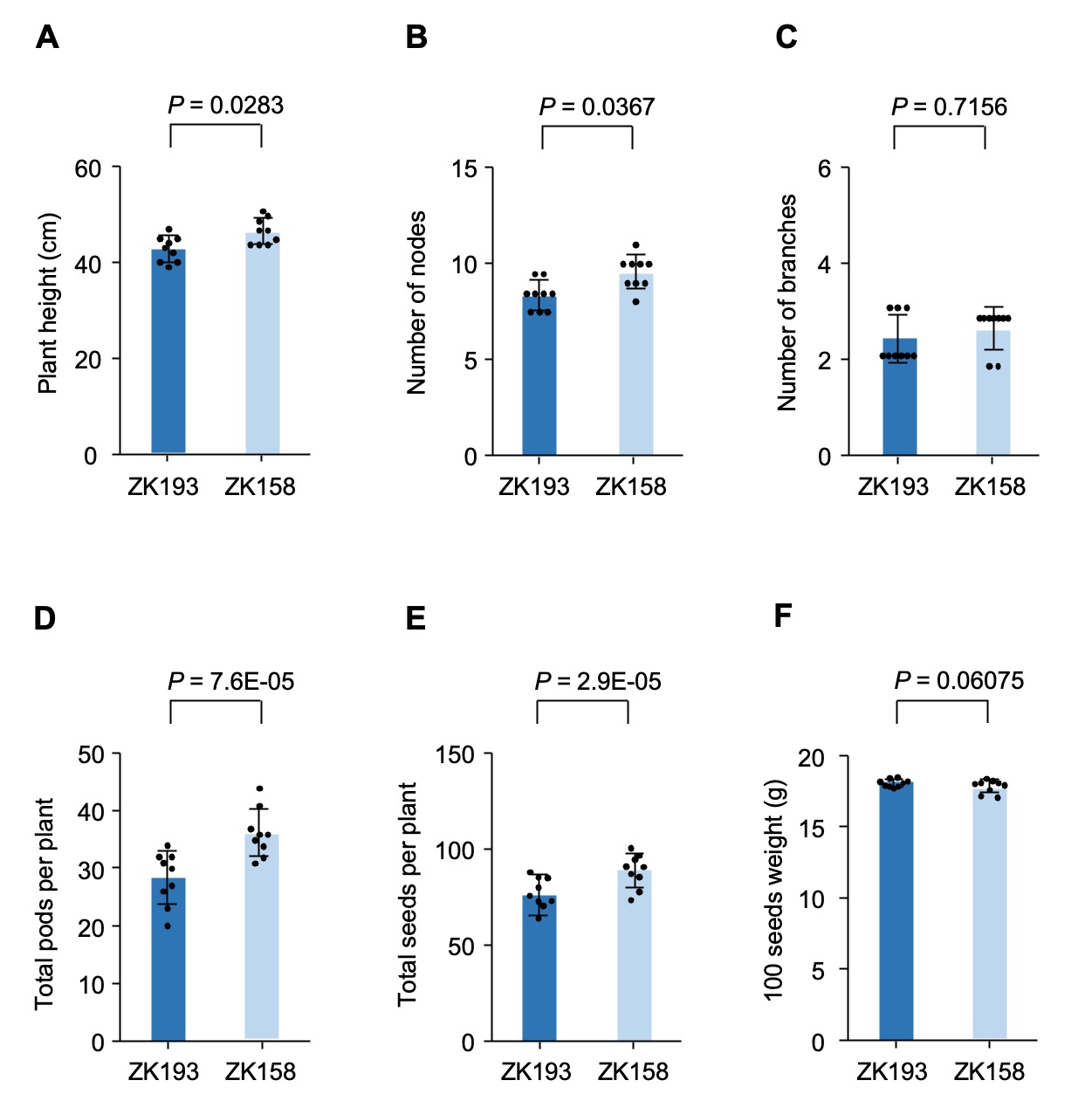

Supplement: Supplementary Figure 1 — Yield related traits of the parent lines. (A–F) Plant height, number of nodes, number of branches, total pods per plant, total seeds per plant, and 100 seeds weight of ZK193 and ZK158. The plants were grown in standard field under artificially controlled SD (12 h light/12 h dark). All data are given as mean ± s.e.m. (n = 10 plants). Two-tailed, two-sample t-tests were used to generate the P values. [file Image_1.jpeg]

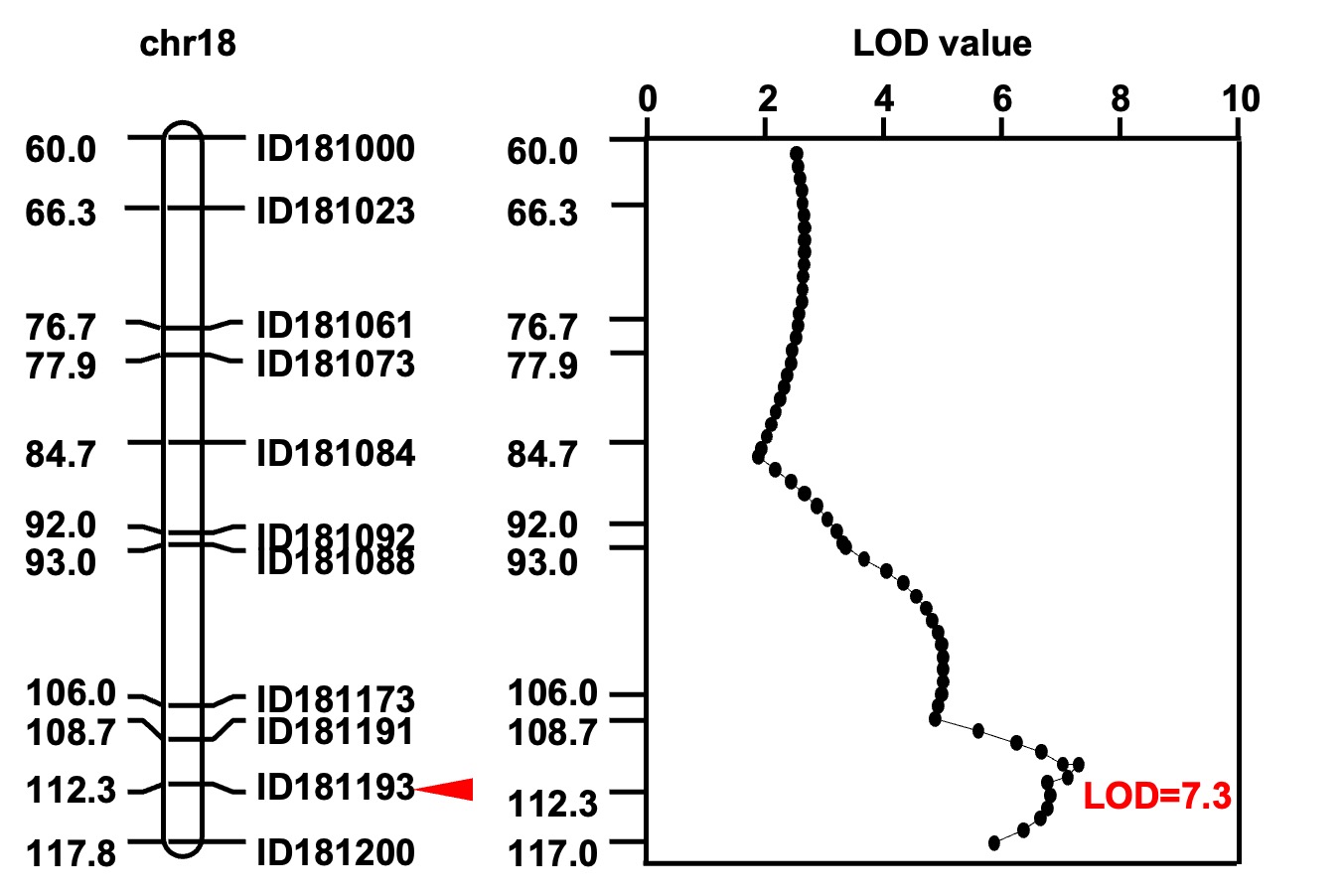

Supplement: Supplementary Figure 2 — Linkage groups containing QTLs for flowering time found in RIL populations. The Linkage groups containing QTLs for flowering time found in RIL populations. The title of linkage groups followed by the chromosome number in parenthesis is indicated at the top. The genetic distance of markers (cM) from the top of each linkage group are given on the left-hand side. The arrows indicate the position of the QTL peaks. [file Image_2.jpeg]

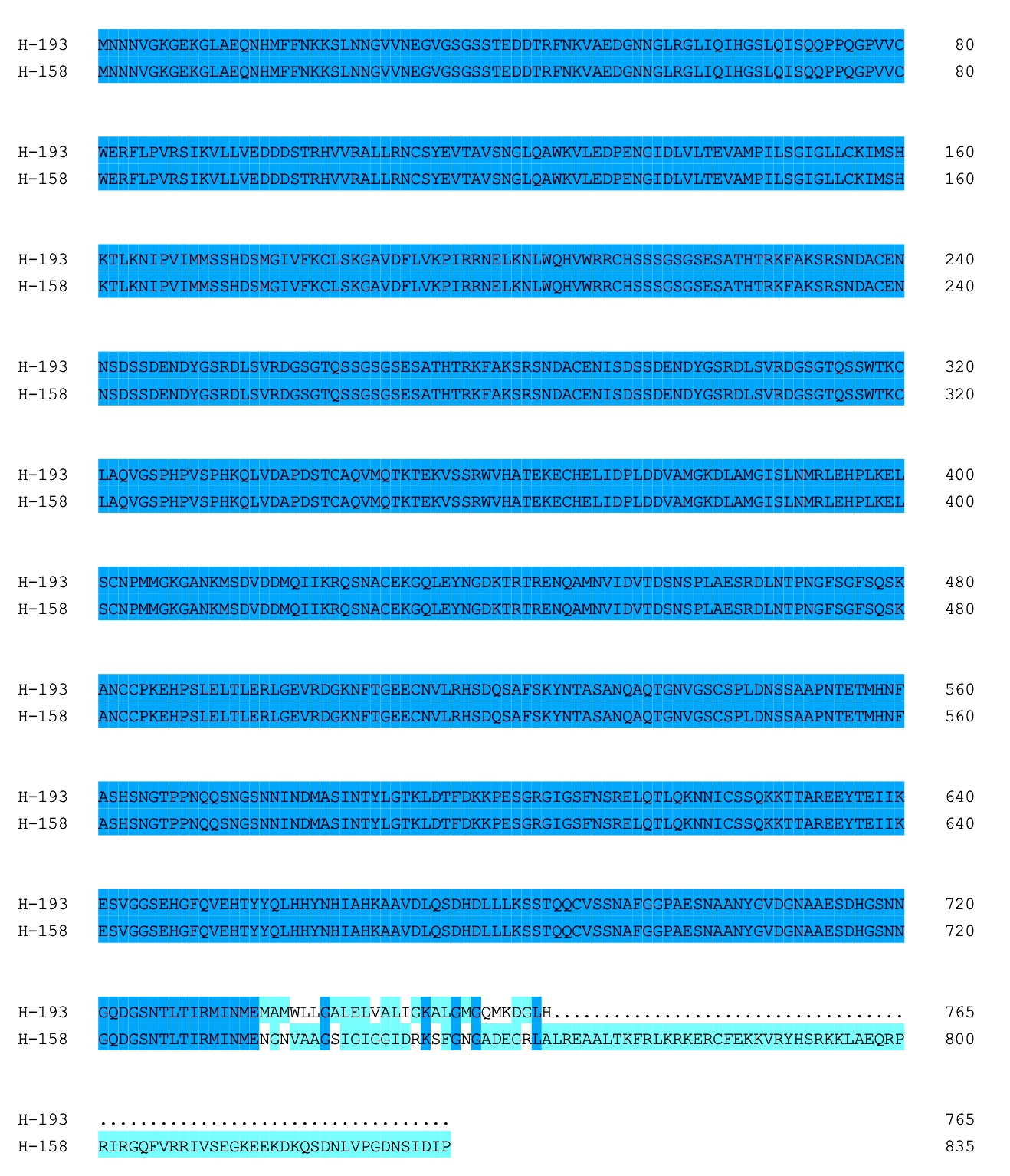

Supplement: Supplementary Figure 3 — Protein sequence alignment of Tof11 alleles. Similarity of the predicted amino acid sequence of Tof11 homologs. The sequences were extracted from the Zhonghuang 13 genome database. [file Image_3.jpeg]

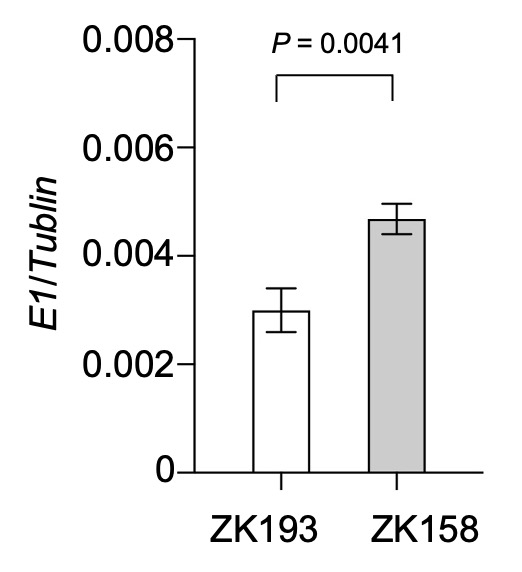

Supplement: Supplementary Figure 4 — The expression level of E1 in ZK193 and ZK158. Expression level of E1 in the NILs. Soybean Tublin was used as an internal control. Data are means ± s.e.m (n = 3). A Student’s t-test was used to generate the P values. [file Image_4.jpeg]

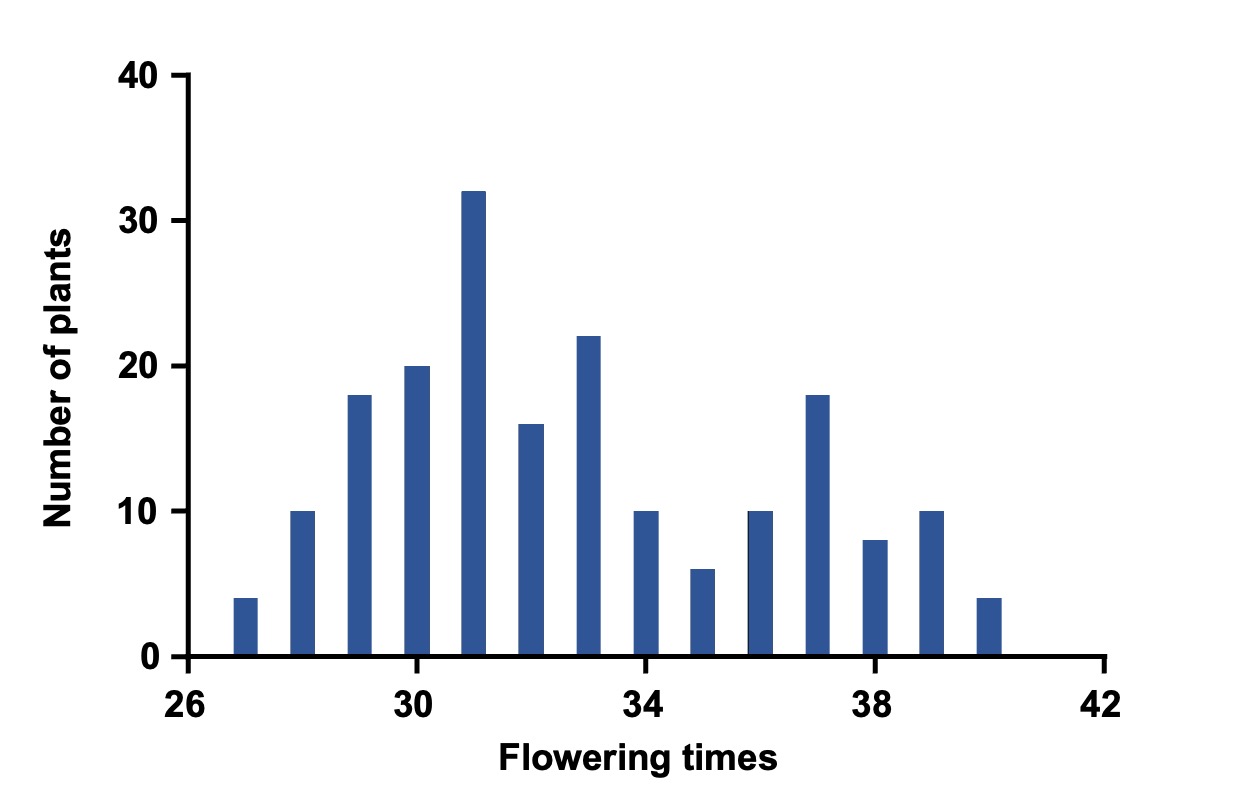

Supplement: Supplementary Figure 5 — Frequency distribution of flowering times in LJ18 residual heterozygous lines (RHL) populations. [file Image_5.jpeg]

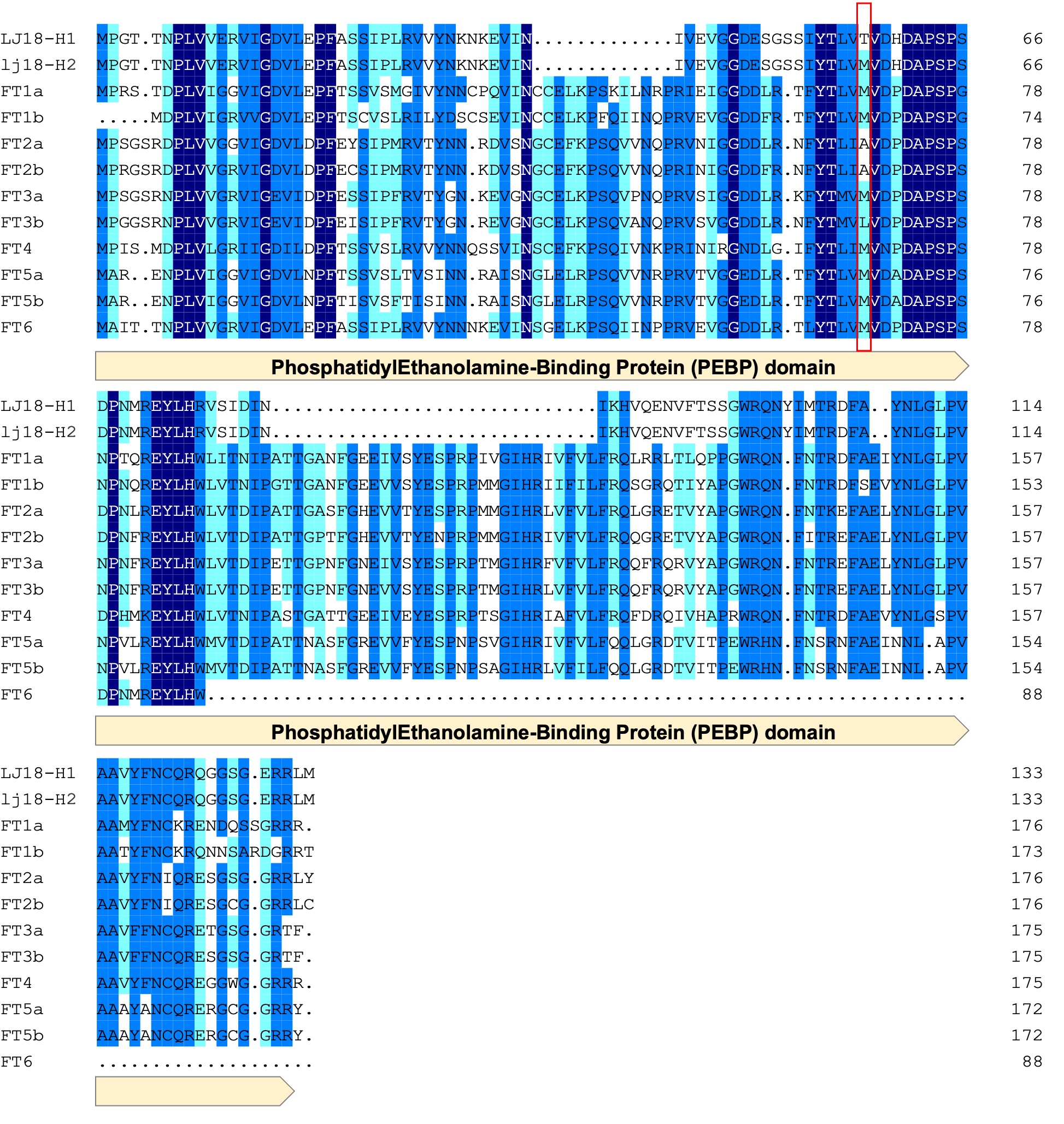

Supplement: Supplementary Figure 6 — Protein sequence alignment of FT homologs. Similarity of the predicted amino acid sequences of FT homologs in soybean. The sequence of LJ18 (Glyma.18G298800), FT1a (Glyma.18G298900), FT1b (Glyma.18G299000), FT2a (Glyma.16G150700), FT2b (Glyma.16G151000), FT3a (Glyma.16G044200), FT3b (Glyma.19G108100), FT4 (Glyma.08G363100), FT5a (Glyma.16G044100), FT5b (Glyma.19G108200), and FT6 (Glyma.08G363200) were obtained from the Williams 82 genome database. Highly conserved amino acids are in dark blue, blue, and light blue depending on the level of identity (darker = higher level). The light-yellow arrows indicate the Phosphatidyl Ethanolamine-Binding Protein (PEBP) domain (predicted by NCBI CD-Search: https://www.ncbi.nlm.nih.gov/Structure/cdd/wrpsb.cgi ). The red box represents the site of core amino acid replacement in the PEBP domain of lj18.1_H2. [file Image_6.jpeg]

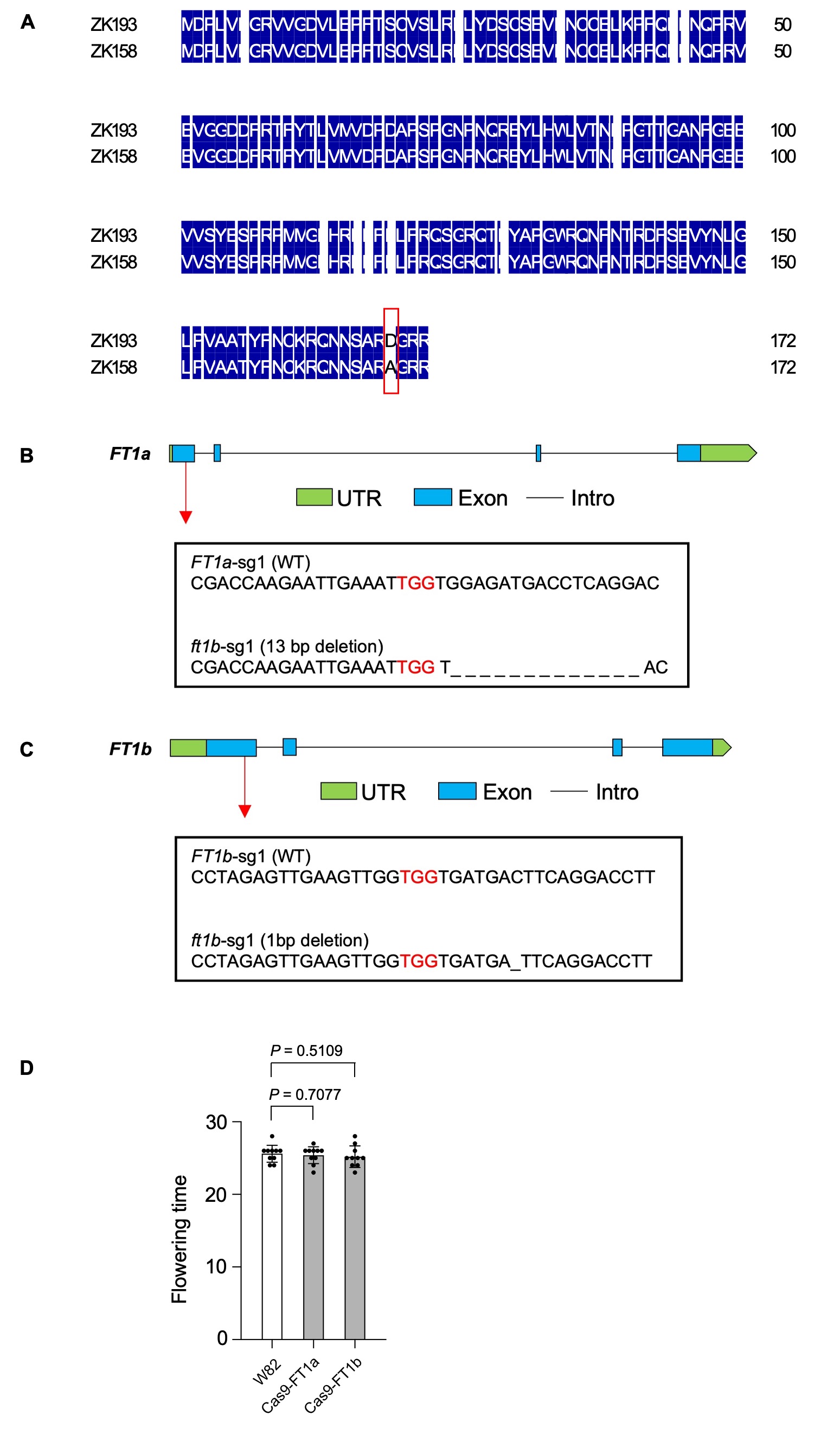

Supplement: Supplementary Figure 7 — Plant material for experiments. (A) The predicted amino acid sequence of Glyma.18G299000 (FT1b) in ZK193 and ZK158, obtained from the Williams 82 genome database. (B, C) Characterization of ft1a and ft1b mutants without Cas9 gene in the T2 generation. Sequences of WT and mutant plants at target sites. Dashes indicate deleted nucleotides. Nucleotides in red indicate PAM. Red arrowheads indicate mutation locations. (D) Flowering time of Cas9-FT1a and Cas9-FT1b under SD (12 h light/12 h dark) conditions. [file Image_7.jpeg]

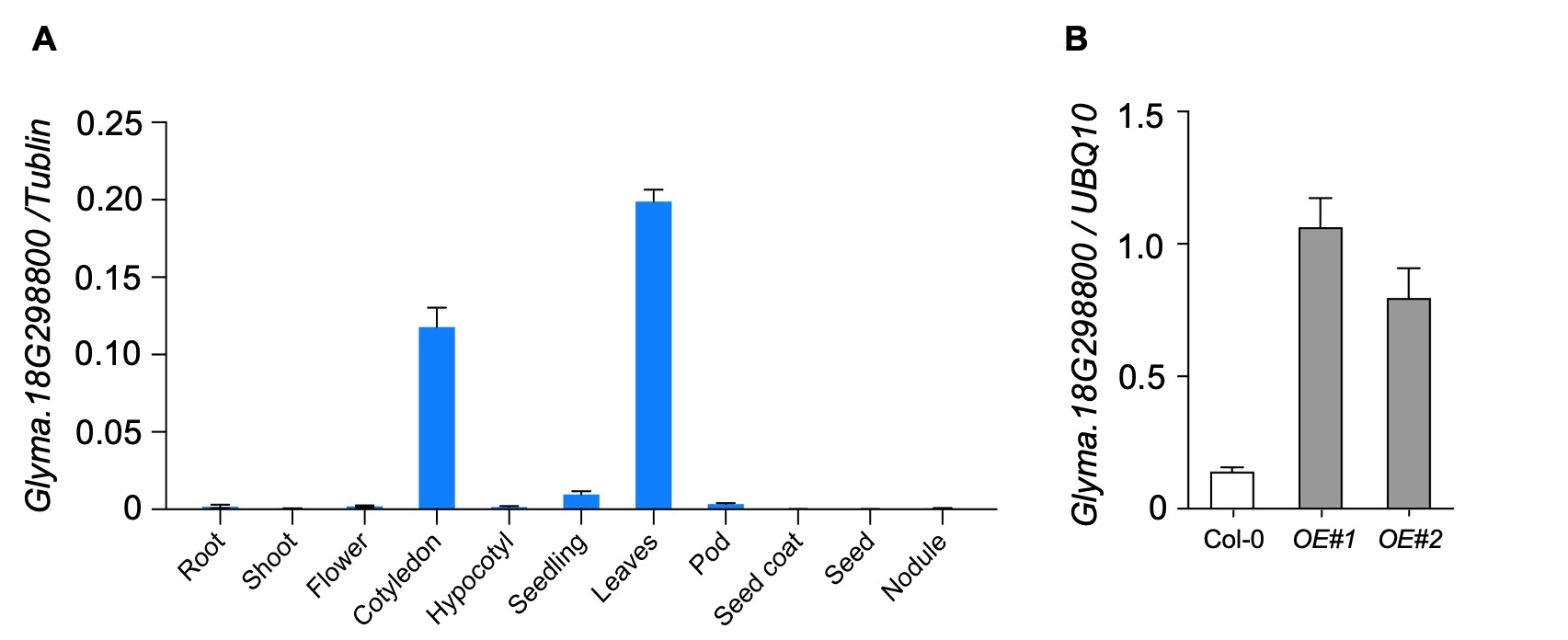

Supplement: Supplementary Figure 8 — Expression pattern of Glyma.18G298800. (A) Expression of Glyma.18G298800 in difference tissues of Williams82 under SD. Soybean Tublin was used as an internal control. Data are means ± s.e.m (n = 3). (B) Expression level of Glyma.18G298800 in the transgenic lines of Arabidopsis. OE, overexpressing. Arabidopsis UBQ10 was used as an internal control. Data are means ± s.e.m (n = 3). [file Image_8.jpeg]
